# Nanomaterials-Based Electrochemical Immunosensors

**DOI:** 10.3390/mi10060397

**Published:** 2019-06-14

**Authors:** Zhenguo Zhang, Yulin Cong, Yichun Huang, Xin Du

**Affiliations:** College of Life Sciences, Key Laboratory of Food Nutrition and Safety, Shandong Normal University, Jinan 250014, China; zhangzhenguo201@163.com (Z.Z.); congyulin824@163.com (Y.C.); Huangyichun9908@163.com (Y.H.)

**Keywords:** nanomaterials, electrochemical immunosensor, carbon, metal, quantum dot

## Abstract

With the development of nanomaterials and sensor technology, nanomaterials-based electrochemical immunosensors have been widely employed in various fields. Nanomaterials for electrode modification are emerging one after another in order to improve the performance of electrochemical immunosensors. When compared with traditional detection methods, electrochemical immunosensors have the advantages of simplicity, real-time analysis, high sensitivity, miniaturization, rapid detection time, and low cost. Here, we summarize recent developments in electrochemical immunosensors based on nanomaterials, including carbon nanomaterials, metal nanomaterials, and quantum dots. Additionally, we discuss research challenges and future prospects for this field of study.

## 1. Introduction

Nanotechnology has gradually become an independent and comprehensive research field since the late 1980s, including microscopy, microelectronics, bioanalytical technology, nanomechanics, and electronics [1,2]. The rapid development of many disciplines, such as biology, has also enabled researchers to obtain deeper understanding regarding the macro and micro worlds. Materials whose structural units are in the nanometer scale (1–100 nm) in at least one dimension or assembled in this range are called nanomaterials or nanostructured materials [3]. When compared with traditional materials, the structure and properties of nanomaterials have changed in essence. At present, nanomaterials have been widely recognized as "the most promising materials in the 21st century" and they are widely used in various fields, such as catalysts, biomedical materials, luminescent materials, insulating materials, and building materials [4,5,6].

When compared with conventional materials, nanomaterials exhibit special properties, including: (1) surface effect: the smaller the diameter of the nanoparticles will have the larger the ratio of the number of surface atoms to the total number of atoms, which in turn causes a sudden change in the properties of the nanoparticles. The concentration of atoms on the surface of the particles will increase the surface energy, as well as the dangling bonds, and can cause the insufficient coordination of the surface atoms, making it easy to combine with other atoms and enhance chemical activity [7,8]; (2) the macroscopic quantum tunneling effect: according to the classical mechanical principle, microscopic nanoparticles can pass through the barrier, because the total kinetic energy of nanoparticles is less than the barrier height. The ability of the nanoparticle to penetrate the barrier is called macroscopic quantum tunneling, which is the basis of future microelectronic devices, including electrochemical immunosensor. When microelectronic devices are further miniaturized, the quantum effects must be considered, because it establishes the limit of size [9]; (3) quantum size effect: the electron energy level of the nanoparticles near the Fermi surface will change from the quasi-continuous energy level to the discrete energy level, or the energy gap becomes wider after the size of the nanoparticle is as small as a certain value, which results in the thermal, electrical, optical, acoustic, magnetic, and superconducting properties of the particles are significantly different from conventional materials [10]. In addition, nanomaterials also have volume effects [11], dielectric effects [12,13], and so on.

Nanomaterials have multiple methods for classification, depending on the discipline and perspective. Nanomaterials can be divided into three categories, according to the dimensions of the basic units. The first category is zero-dimensional nanomaterial, such as quantum dots and atomic clusters, all of which are in the order of nanometers [14,15]. The second is one-dimensional nanometers nanomaterials, such as nanorods and nanowires, which have two dimensions in the three-dimensional space [16,17]. The last category is two-dimensional nanomaterials, such as superlattices and nano-films [18,19]. The methods for preparing nanomaterials have been continuously developed and enriched since the successful development of metal nanoparticles in the 1970s. Owing to the great application potential of nanomaterials, how to prepare nanomaterials with excellent performance, high efficiency, and low cost is still a hot spot for international researchers [20,21]. At present, the main preparation methods of nanomaterials include physical methods, comprehensive methods, and chemical methods. The physical methods include mechanical grinding, evaporation condensation, laser beam, and ion sputtering [22,23,24]. The chemical methods include microemulsion methods, electrochemical deposition method, complex decomposition method, and hydrothermal method [25,26,27]. The comprehensive method mainly contains the laser gas phase synthesis method, ultrasonic chemical method, and plasma enhanced chemical deposition method [28,29].

**Figure 1:**
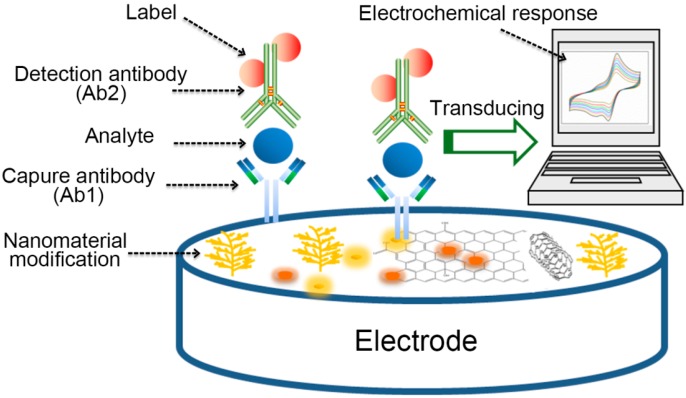
A schematic representation of electrochemical immunosensor.

The electrochemical immunosensor based on antigen-antibody immunoreactivity is the largest class of electrochemical methods being used for protein analysis in biological research and clinical testing. Traditional immunoassays include enzyme-linked immunosorbent assays [30], immunoblot analysis [31], radio immunoassays, and immune electrophoresis [32,33], which are mainly based on the changes of signal that are generated by specific binding of target proteins to known antibodies [34]. Electrochemical immunoassay, as a currently mature technique, has displayed advantages with high sensitivity of electrochemical technology and high specificity of immune recognition reaction, so it can be used in many fields [35,36]. Electrochemical immunoassays can be broadly classified into label-free sensor and labeled sensor. The signal amplification is the core of the preparation electrochemical biosensor, which was realized by nanomaterials modification on the surface of electrode. The immunosensor can selectively identify the analyte by the capture antibody. After that, the label-free electrochemical immunoassay determines the concentration of the analyte by directly measuring the antigen-antibody specific recognition of the change in the electrochemical signal that is generated after binding. The electrochemical impedance signal can effectively reflect various physical and chemical processes that occur on the surface of the electrode, and thus it is often applied for the detection of proteins. The sandwich-type electrochemical immunosensor also used the Ab2, which is often labeled by electrochemical probe and nanomaterials. Last, as the main body of the signal converter, the electrode can derive the identification signal that is generated on the surface of the electrode and convert it into an electrical signal, including current, voltage, and resistance, which can be measured and analyzed in order to achieve the qualitative or quantitative analysis of the analysis target. Figure 1 shows the operating principle of electrochemical immunosensor.

## 2. Nanomaterials Based Electrochemical Immunosensor

The use of nanomaterials to modify the electrode surface can not only significantly enhance the ability of the electrode to transport electrons, but also improve the adsorption capacity of the electrode surface to bioactive substances, and then shorten the detection time thanks to the advantages of good biocompatibility, high surface reactivity, and large specific surface area of nanomaterials. There are currently three types of nanomaterials that are mainly used in the modification of electrode of electrochemical immunosensor: carbon nanomaterials, metal nanomaterials, and quantum dots [37,38]. 

### 2.1. Electrochemical Immunosensor Based on Carbon Nanomaterials

Adams first proposed carbon-based electrodes in 1958. In addition to the reproducibility of working electrodes by simple sanding, carbon or carbon-based materials have many other advantages, including ease of preparation and catalysts. Carbon nanomaterials can be uniformly dispersed in aqueous solution with good stability and low electrical resistance. With the use of carbon nanomaterials (graphene, carbon nanotubes, fullerenes, etc.) in the electrochemical immunosensor, the signal-to-noise ratio of the reaction occurring between the interfaces and the sensitivity of the biosensor is improved. Table 1 summarizes the carbon nanomaterials-based electrochemical immunosensors.

**Figure 2:**
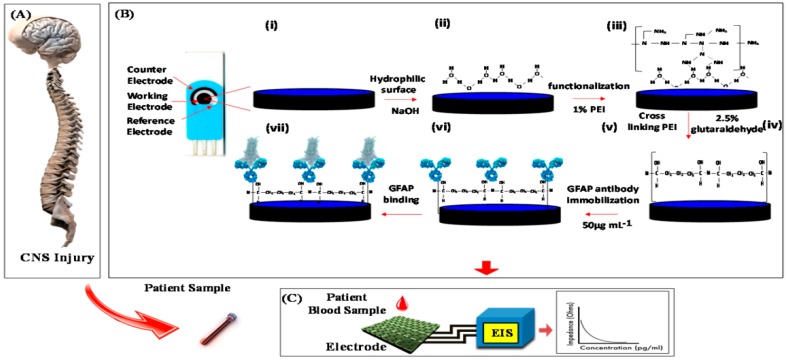
Schematic representation of the modification of a graphene electrode surface, sample collection, and electrochemical detection of glial fibrillary acidic protein (GFAP): (**A**) Blood collected from central nervous system (CNS) injury patient, or samples are prepared in artificial cerebrospinal fluid and in blood serum. (**B**) Stages of surface modification for binding the GFAP antibody to the functionalized graphene electrode: (i) Bare graphene electrode, (ii) sodium hydroxide (NaOH) treatment for creating hydrophilic surface, (iii) functionalization of polyethylenimine (PEI), (iv) activation of the surface with glutaraldehyde, (v) Schiff base, (vi) immobilization of GFAP antibody, (vii) blocking unbounded sites, and (viii) immunosensor ready for the detection. (**C**) Detection of electrochemical impedance spectroscopy (EIS) response of the immunosensor for GFAP detection. Reproduced with permission from [45].

Graphene, which is a single atomic thick sheet of graphite composed of sp^2^ bonded carbon, has been widely used in electrochemical immunosensor since its first discovery in 2004 [39,40,41]. Pham et al. [42] developed a label-free electrochemical immunosensor for the detection of microRNAs (miRNA) while using a conducting polymer/reduced graphene oxide (CP/RGO)-modified electrode to detect miR-141 (a prostate biomarker) and miR-29b-1 (a lung cancer biomarker). They employed two specific RNA−DNA antibodies to recognize miRNA−DNA heteroduplexes and square wave voltammetry to detect the redox signal in order to verify its selectivity. Whereafter, this group reported another electrochemical immunosensor for the determination of these two miRNAs based on RGO-carbon nanotubes modified gold electrode [43]. The secondary antibody was labeled by horseradish peroxidase (HRP) and hydroquinone was used as signal molecular. The performance of the prepared immunosensor was more excellent than classical optical detection. The study of the performance of immunosensors in the above papers was focused on selectivity and sensitivity, so the reproducibility and stability were not discussed. Hu et al. [44] used streptavidin-functionalized nitrogen-doped graphene (NG) for the first time to fabricate a highly sensitive electrochemical immunosensor for the detection of tumor markers. The bio-functionalized NG not only showed excellent electrochemical performance, but it also adsorbed more antibodies. Combining the secondary antibody labeled by HRP, the prepared CEA immunosensor revealed satisfactory results when being applied to the detection in human serum samples. Recently, Sanati-Nezhad et al. [45] reported an immunosensor that was based on polyethylenimine (PEI) coated graphene screen-printed electrode for the highly sensitive determination for glial fibrillary acidic protein (GFAP), which is a marker of central nervous system injury (Figure 2). The label-free and quantitative detection of GFAP was realized through the method of electrochemical impedance spectroscopy. This prepared immunosensor could be used for the rapid monitoring of central nervous system (CNS) injury in clinic.

**Figure 3:**
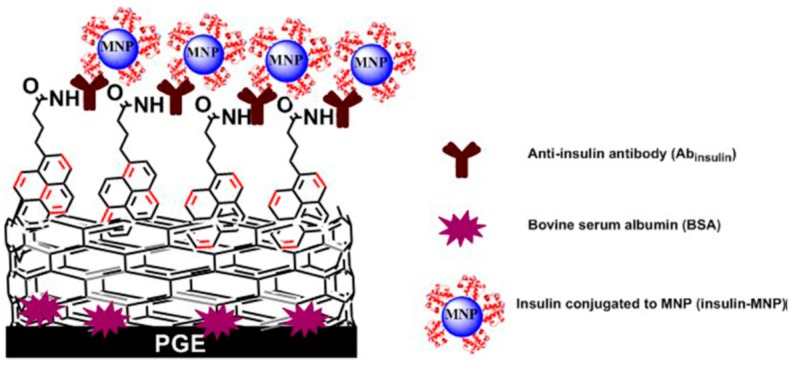
Schematic design of immunosensor for insulin detection in patient serum. Reproduced with permission from [49].

Carbon nanotubes (CNTs), which are also known as Buckytubes, were composed of well-ordered cylinders of sp^2^-hybridized carbon atoms that were first discovered by S.Iijima in 1991 and belong to the fullerene carbon system. The hexagonal carbon atoms were first to constitute the graphene, which is curled into several layers at a certain spiral angle of coaxial nano-scale round tubes. The two ends of the CNTs are basically sealed, whose diameter and length are generally in the range of 2 to 100 nm and micrometers, respectively. According to the number of layers constituting the sheet structure of CNT, the CNTs mainly include two types of single-walled carbon nanotubes (SWCNTs) and multi-walled carbon nanotubes (MWCNTs) [46]. SWCNTs, which are also known as fullerenes tubes, are hollow cylinders that crimped from a layer of graphene sheets. The MWCNTs are composed of two or more layers of coaxial circular tubular graphene sheets, and a fixed distance of about 0.34 nm that was caused by van der Waals forces is maintained between the layers [47]. F. Rusling et al. [48] reported an electrochemical sandwich immunosensor that was based on single wall carbon nanotube (SWNT) forests with attached capture antibodies (Ab1) and multiwall carbon nanotubes-HRP labeled second antibody to detect IL-6. The prepared immunosensor could accurately measure the secreted IL-6 from a wide range of HNSCC cells, which is in agreement with standard enzyme linked immunosorbent assays (ELISA). Krishnan et al. [49] designed a voltammetric immunosensor for the diagnosis of type 1 and type 2 diabetic disorders that were based on multi-walled carbon nanotube-pyrenebutyric acid frameworks on edge plane pyrolytic graphite electrodes (PGE/MWNT/Py) attached anti-insulin antibody for the first time (Figure 3). Poly(acrylic acid)-functionalized magnetite nanoparticles (MNP, 100 nm hydrodynamic diameter) was applied to label Ab2. The electrochemical signal of the immunosensor was decreased when it was taken in the electrolyte solution.

**Figure 4:**
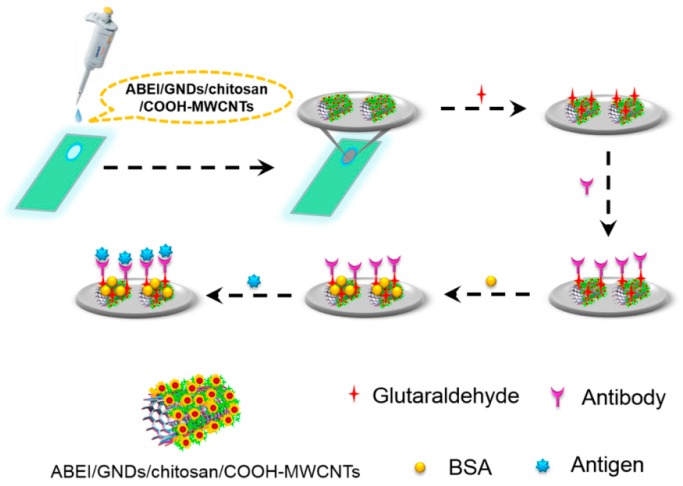
Schematic description for label-free N-terminal pro-brain natriuretic peptide (NT-proBNP) immunosensor based on ABEI/GNDs/chitosan/COOH-MWCNTs. Reproduced with permission from [50].

Bian et al. [50] developed a novel electrochemiluminescence (ECL) immunosensor for the detection of N-terminal pro-brain natriuretic peptide (NT-proBNP) based on N-(aminobutyl)-N-(ethylisoluminol) (ABEI)-functionalized MWCNTs/gold nanodots (ABEI/COOH-MWCNTs/chitosan/GNDs) hybrid modified ITO electrode, as shown in Figure 4. The principle of the immnosensor was also that the signal of ECL would decrease when NT-proBNP was captured on the surface of electrode by its antibody. The prepared immunosensor also displayed satisfactory performance when quantifying the NT-proBNP in practical plasma samples, which revealed that the ABEI/COOH-MWCNTs/chitosan/GNDs nanomaterial was a superior electrochemical sensing platform. Recently, Omidi et al. [51] constructed a simple electrochemical immunosensor for the determination of carcinoma antigen 125 (CA125) based on chitosan-gold nanoparticle/multiwall carbon nanotube/graphene oxide (CS-AuNP/MWCNT/GO) platform. The lactate oxidase is applied as the single-enzyme label in electrochemical immunosensor for the first time. The immunosensor was realized in the detection of CA125 in human serum.

**Figure 5:**
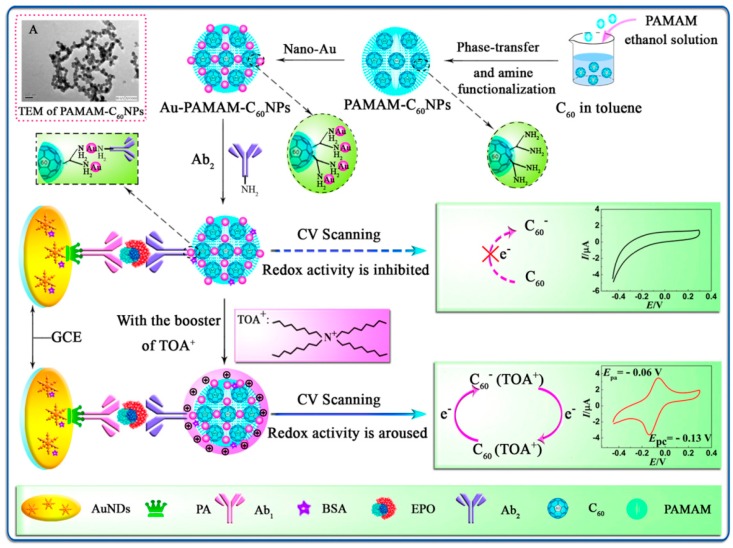
Schematic illustration of the immunosensor preparation process and possible mechanism of electrochemical reaction. Reproduced with permission from [51].

Yuan et al. [52] employed the inner redox activity of fullerene (C_60_) to construct an electrochemical immunoassay for doping detection (Figure 5). They first decorated the C_60_ nanoparticles by polyamidoamine (C_60_NPs-PAMAM) to enhance its hydrophilicity and modification site. After that, the AuNPs were linked on C60NPs-PAMAM and used as nanoprobe to label antibodied. C_60_NPs-Au-PAMAM would produce redox electrochemical signal when tetraoctylammonium bromide (TOAB) aroused the inner redox activity of C_60_. Recently, Kumar et al. [53] designed a novel electrochemical redox platform that was based on sesamol-quinone/carbon nanoblack modified platform (GCE/CB@Ses-Qn). In this work, they compared the cyclic voltammetry curve (CV) responses of different carbon nanomaterials modified electrodes, including multi-walled carbon nanotube (MWCNT), activated charcoal, double-walled carbon nanotube, GO, single-walled carbon nanotube, carboxylic acid-functionalized MWCNT, and CB. The results demonstrated that the sensor that was based on CB displayed the highest current signal and excess surface values of sesamol loading. In the end, the platform was used to fabricate an electrochemical immunosensor for the detection of white spot syndrome virus while using HRP-linked secondary antibody.

As an important part of nanomaterials, metal nanomaterials have the characteristics and properties of nanomaterials, such as macroscopic quantum tunneling, quantum size effects, and surface effects [54,55,56]. It has been used in various fields, such as biosensors and electronic products. The main preparation methods of metal nanomaterials include physical preparation methods and chemical preparation methods [57,58]. The physical methods mainly include evaporation condensation, sputtering, and mixed plasma method; the chemical methods mainly include phase chemical synthesis, chemical reduction method, electrochemical method, and liquid phase chemical reduction method. Table 2 summarizes the metal nanomaterials-based electrochemical immunosensors.

At present, gold (Au), platinum (Pt), silver (Ag), palladium (Pd), titanium (Ti), and other metal nanomaterials have been widely used in the active modification of the electrode surface to prepare immunosensor. Main functions of metal nanomaterials can be summarized, as the following. The first function is that metal nanomaterial can be used as a signal molecule to label the bioactive substances [59,60]. In addition, metal nanomaterial can be used as an excellent carrier to adsorb bioactive substances because of its large specific surface area and biocompatibility [61]. Metal nanomaterials can also directly participate in the reaction of the electrode surface as a reactant or catalyst. Many metal nanomaterials (such as Cu and Pt) are themselves high-quality electrochemical reaction substrates or catalysts, which not only increase the peak value of the reaction current, but also reduce the potential of the reaction and amplify the signal intensity. Last, the metal nanomaterials can enhance electron transport as an enzyme mediator for electron transport [62]. For example, when the enzyme sensor is working, the active center of the enzyme is often buried inside the enzyme, which is not conducive to the electron transfer between the catalytic active center and the electrode surface, which thereby affects the sensitivity and detection time of the sensor. The metal nanoparticles have strong electrical conductivity and it can serve as an electron mediator between the enzyme and the electrode surface. The experiments have shown that metal nanoparticle-modified electrodes can significantly accelerate the electron transport speed.

Bai et al. [63] completed the simultaneous detection of prostate specific antigen (PSA) and a-fetoprotein (AFP) based on Au nanoparticles modified polymer brush (poly (acrylonitrile-g-glycidyl methacrylate)) (AuNPs/PGMA-g-PAN). The antibodies of PSA and AFP were labeled by anthraquinone-2-carboxylic acid (Aq) and ferrocenecarboxylic acid (FeC-COOH), respectively, and the developed immunosensor demonstrated good performance, even if in clinical serum analysis. Wei et al. [64] developed an immunoassay for the detection of carcinoembryonic antigen (CEA) based on Ag nanoparticles-molybdenum disulfide-reduced graphene oxide (Ag/MoS_2_/rGO) nanocomposites platform, which could catalyze hydrogen peroxide (H_2_O_2_) and produce the electrochemical signal. The prepared immunosensor displayed a low detection limit (1.6 fg/mL) while using amperometric i-t curve with acceptable reproducibility, selectivity, and excellent stability. Following the report, Wei et al. [65] employed Pd nanocubes functionalized magnetic graphene sheet (Pd–Fe_3_O_4_-GS) and silicon dioxide (SiO_2_) to label the secondary antibodies (Ab2) of the target detector. The developed immunosensor displayed an ultrasensitive and specific detection of human immunoglobulin G (IgG), owing to the superior electrochemical catalytic ability to H_2_O_2_ of the matrix composed by which used to immobilized the primary antibodies (Ab1).

**Figure 6:**
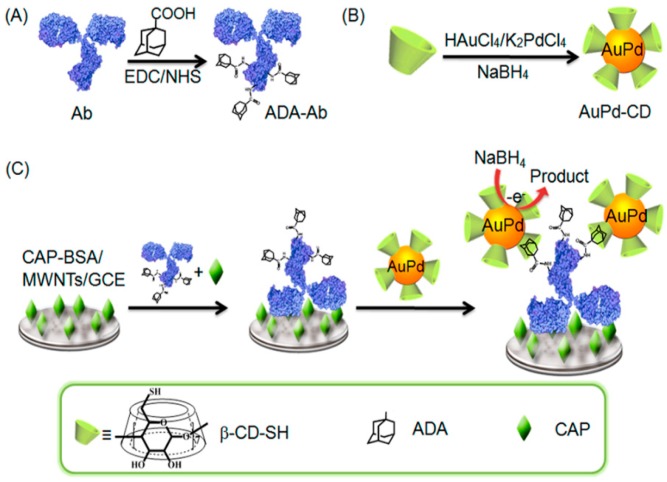
Preparation of (**A**) ADA−Ab conjugate and (**B**) β-CD-functionalized AuPd bimetallic nanoparticles, and (**C**) electrochemical immunoassay procedure for the detection of a small molecule. Reproduced with permission from [66].

**Figure 7:**
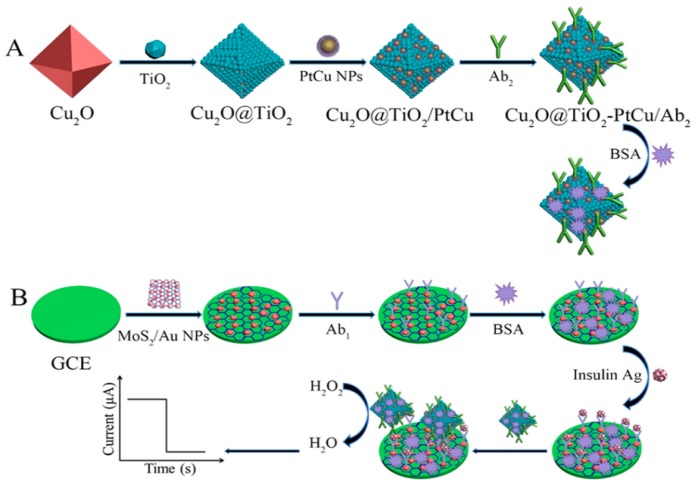
(**A**) Preparation process of PtCu NPs-Cu_2_O@TiO_2_-Ab2 and (**B**) the fabricated process of the proposed sandwich-type immunosensor. Reproduced with permission from [47].

Bimetallic or polymetallic nanomaterials are increasingly popular among researchers for preparation of immunosensor, owing to the synergistic effects of metal composite nanomaterials [66,67]. For example, Ju et al. [68] synthesized β-CD functionalized gold−palladium bimetallic nanoparticles (AuPd−CD) in aqueous solution and used it as a platform to detect the adamantine, which was labeled with antibody (ADA-Ab), as shown in Figure 6. AuPd nanoparticles could catalyzed NaBH_4_ and produce an ultrasensitive response to the target chloramphenicol (CAP). The host−guest interaction strategy between CD and ADA provided a universal labeling approach for the ultrasensitive detection of small molecule targets. Recently, Wei et al. [69] fabricated dendritic platinum−copper nanoparticles (PtCu NPs), which were loaded on titanium dioxide octahedral composites (Cu_2_O@TiO_2_-NH_2_) in order to realize the signal amplification of the insulin electrochemical immunosensor (Figure 7). The PtCu NPs-Cu_2_O@TiO_2_ was used to label Ab2. The immunosensor acquired satisfactory results when detecting insulin in human serum based on the platform of AuNPs /MoS_2_ loaded Ab1.

**Figure 8:**
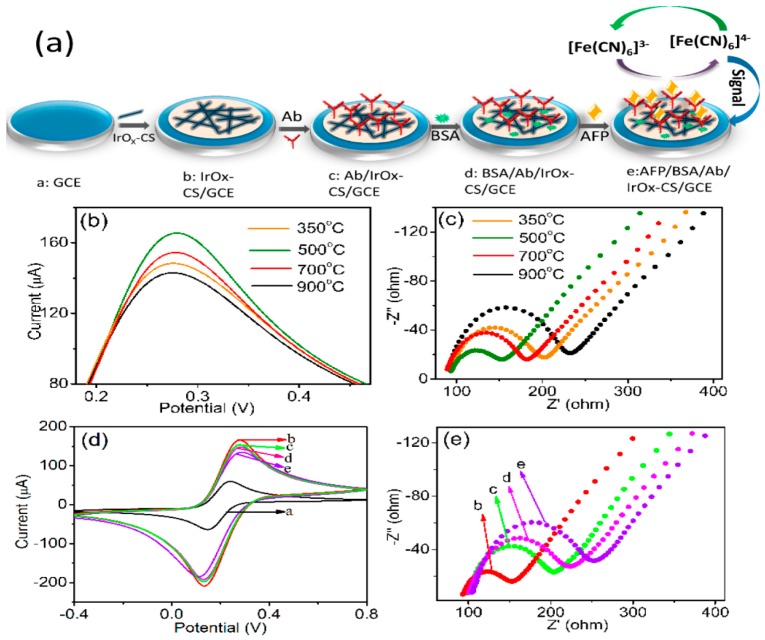
(**a**) A schematic diagram of the detailed preparation process of an IrOx-nanofiber-modified immunosensor. (**b**) cyclic voltammetry curve (CV) and (**c**) EIS spectra of different IrOx-nanofiber-modified immunosensors. (**d**) CV and (**e**) EIS spectra of the fabrication progress of IrOx-nanofiber-modified (annealed at 500 °C) immunosensor in pH 7.4 PBS solution containing 5.0 mM [Fe(CN)_6_]^3−/4−^, where the letters labeling the spectra correspond to the diagram labels in panel a. Reproduced with permission from [71].

In addition to metal nanoparticles, metal oxides are also a large class of excellent nanomaterials for the construction of electrochemical immunosensors [70]. For example, Song et al. [71] fabricated a label-free immunosensor for the detection of cancer biomarker α-fetoprotein (AFP), while taking advantage of iridium oxide (IrOx, 0 ≤ x ≤ 2) nanofibers that were prepared by a simple one-spinneret electrospinning method (Figure 8). The IrOx nanofibers displayed superior performance and provided a highly stable matrix for the conjugation of chitosan (CS), which facilitate the immobilization of antibody. The immunosensor has also shown satisfactory results when determining AFP in human serum. Wei et al. [72] chose the amino functionalized Co_3_O_4_@MnO_2_-thionine (Co_3_O_4_@MnO_2_-Th) as secondary label, which could greatly enhance the electrochemical response signal in order to develop a sandwich-type electrochemical immunosensor for the determination of alpha fetoprotein (AFP). Titanium oxide nanoclusters functionalized nitrogen-doped reduced graphene oxide (TiO_2_-NGO) decorated by Au@Pd holothurian-shaped nanoparticles (Au@Pd HSs) were used to modify the surface of electrode for the detection of human epididymis specific protein 4 antigen (HE4 Ag) [73]. The perfect catalytical activity to H_2_O_2_ of Au@Pd HSs could endow the superior performance to the developed electrochemical immunosensor.

**Figure 9:**
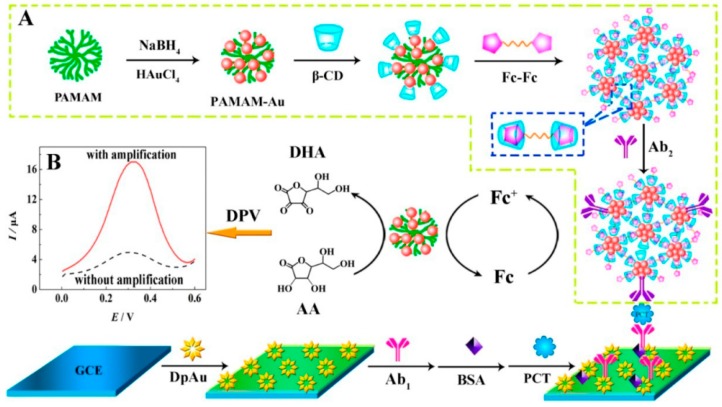
Schematic diagram of fabrication of electrochemical immunosensor for detection of procalcitonin (PCT). (**A**) Preparation procedure of Fc-Fc/β-CD/PAMAM-Au-labeled Ab2 bioconjugates. (**B**) Comparative DPV signals with and without amplification. Reproduced with permission from [74].

Researchers have found that the morphology and structure of metal nanomaterials also have an important impact on their performance with the wide application of metal nanomaterials in electrode modification. Therefore, how to realize the controllability of the size, structure, and morphology of metal nanomaterials, as well as the application of metal nanomaterials with special structural morphology to the field of biosensing, has attracted great interest from researchers in recent years. Most metal nanomaterials with special morphology in the fabrication of a sensor used its own catalytic capacity to the targets, for example, hydrogen peroxide, glucose, and ascorbic acid. There are also have several reports regarding electrochemical immunosensors that are based on mental nanomaterials with special morphology. For example, Yuan et al. [73] synthesized netlike Au nanostructure using β-cyclodextrins and poly(amidoamine) as platform (Figure 9). The prepared Fc-Fc was absorbed by the β-CD to form Fc-Fc/β-CD/PAMAM−Au, which was attached to the Ab2. The netlike nanostructure could attach large amounts of the β-CD and Ab2; meanwhile, the nanomaterial will amplify the current signal. The constructed immunosensor was used to detect the procalcitonin (PCT) with perfect performance.

**Figure 10:**
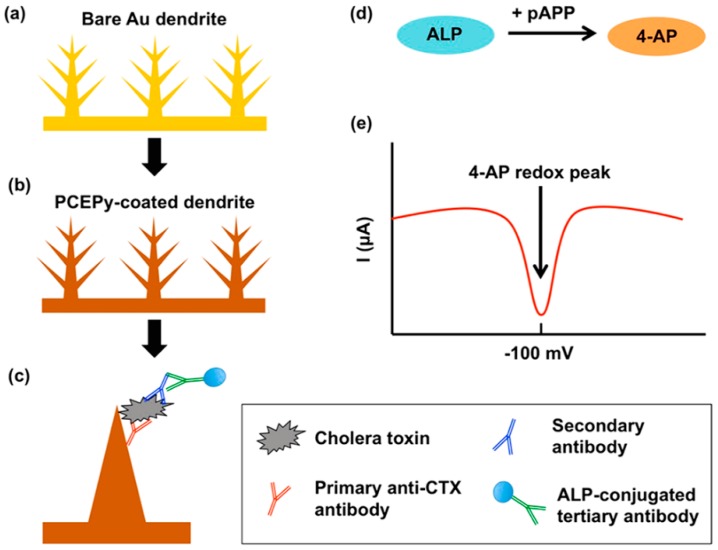
Schematic overview of the dendrite-based on-chip enzyme linked immunosorbent assays (ELISA). (**a**) Dendrites grown on a planar gold substrate. (**b**) Dendrites coated in a film of poly(2-cyanoethyl)pyrrole (PCEPy). (**c**) Primary antibody to cholera toxin (CTX), tethered to PCEPy-coated dendrites via electrostatic interactions. Secondary anti-CTX antibody, and tertiary antibody are added, with wash steps between each. (**d**) Alkaline phosphatase (ALP) conjugated to the tertiary antibody reacts with the substrate p-aminophenyl phosphate (pAPP) to form 4-aminophenol (4-AP), which (**e**) oxidizes at −100 mV vs a pseudo Ag/AgCl reference electrode. This redox peak is proportional to the amount of CTX in the sample. Reproduced with permission from [73].

Furthermore, metal nanomaterials with the morphology of nanowires, nanorod, or dendritic structure as one-dimensional nanometers nanomaterials are applied in the construction of electrochemical immunosensor. For example, the Ag nanowires were prepared for capturing Ab1 and thionine(TH)-doped mesoporous ZnO nanostrawberries (MP-ZnO) were used to immobilize the HRP-anti-IgG by Wang et al. [75]. The super conductivity of silver nanowires enhanced the electrochemical signal, which demonstrated the advantage of one-dimensional (1D) nanomaterial. Zhu et al. [76] used platinum nanowire inlaid globular SBA-15 (Pt NWs@g-SBA-15/Thi) as the signal probe and double-deck gold film (D-Au film) as electrode modified nanomaterials to develop an electrochemical for detecting hepatitis B surface antigen (HBs Ag). The nanomaterial could accelerate the electron transfer on the electrode interface, owing to the tunneling effect between the two Au films. The advantage of Pt NWs@g-SBA-15/Thi is that the nanocomposite could reduce the spatial limitation and then load more Ab2 and provide abundant catalytically active sites for analyst. Recently, Chiles et al. [77] reported an on-chip electrochemical immunosenor in order to test the cholera toxin subunit B(CTX) based on a dendritic gold architecture that was modified through poly(2-cyanoethyl)pyrrole (PCEPy), which was synthesized by electrodeposition for 20 min. in a solution of 30 mM HAuCl_4_ (Figure 10). The surface area of dendritic gold electrode was 18× greater than a planar gold surface which allowing for a higher sensitivity. The prepared immunosensor meted the demand for point-of-care for the diagnosis of cholera, which demonstrated the value of the reported dendritic gold architecture.

### 2.2. Electrochemical Immunosensor Based on Quantum Dots

Quantum dot (QD) that was composed of III-V atoms (such as GaAs, InP) or II-VI atoms (such as CdTe, CdS), also known as semiconductor nanocrystal, is a uniform inorganic nanoscale particle that is generally within 10 nm in diameter with a core/shell structure [78,79]. QD is an excellent tool to prepare an electrochemical immunosensor, because it has all of the special properties of nanomaterials, including quantum size effects, surface effects, and unique characteristic of high electron density [80,81]. Several researchers have reported the use of voltammetry to detect QDs as electrochemical labels, taking advantage of their redox properties [82,83,84]. Table 3 summaries the QD nanomaterials-based electrochemical immunosensors.

**Figure 11:**
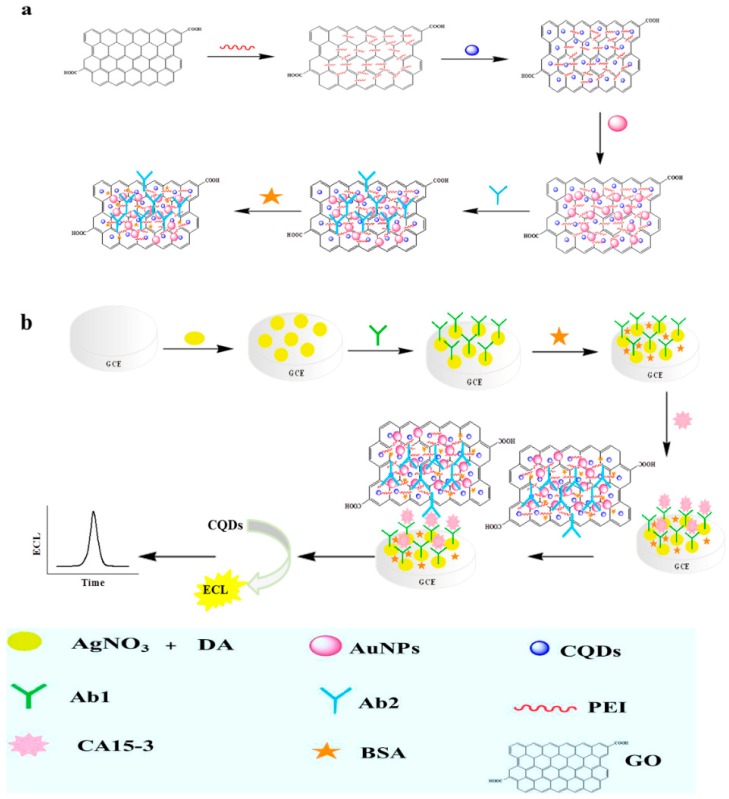
(**a**) Immobilization of AuNPs, CQDs, and Ab2 on the polyethylenimine-GO (PEI-GO) Matrix. (**b**) Fabrication Process of Proposed electrochemiluminescence (ECL) Immunosensor. Reproduced with permission from [84].

A novel magneto-controlled electrochemical immunosensor was constructed for the sensitive detection of low-abundance protein (IgG1) with a sandwich-type assay that was based on CdS QD-doped bovine serum albumin (QD-BSA) and IgG1-functionalized magnetic bead [85]. The electrochemical signal was studied while using anodic stripping voltammetric analysis of cadmium ion released by acid from quantum dot. Lin et al. [86] developed an electrochemical immunosensor for the detection of organophosphorylated butyrylcholinesterase (OP-BChE), which is a specific biomarker for exposure to toxic organophosphorus agents. QD was employed to tag anti-BChE antibody in order to amplify the signal. The prepared immnosensor displayed a highly selective and sensitive response to the target. Gong et al. [87] reported electrochemical immunosensors for the detection of prostate specific antigen (PSA) that was based on QD functionalized graphene sheets (GS-QD). The immunosensor was capable of detecting PSA in serum samples. Employing carbon QDs-graphene oxide-PEI-Au nanohybrid (CQDs-PEI-GO/AuNPs) as a probe to sense carbohydrate antigen 15-3 (CA15-3) was used to develop a novel electrochemiluminescence immunosensor (Figure 11) [88]. The Ab1 was linked on Ag nanoparticles and polydopamine (AgNPs-PDA), which had large surface area. The immusensor exhibited excellent performance, owing to the synergistic effect of the nanocomposite materials. A miniaturized electrochemical immunosensor was established by using 8-channel screen-printed carbon arrays and IgG labeled with CdSe/ZnS QDs for the detection of anti-transglutaminase antibodies (a celiac disease biomarker, tTG) in human sera [89]. The developed miniaturized electrochemical immunosensor is easier to use for clinic detection.

**Figure 12:**
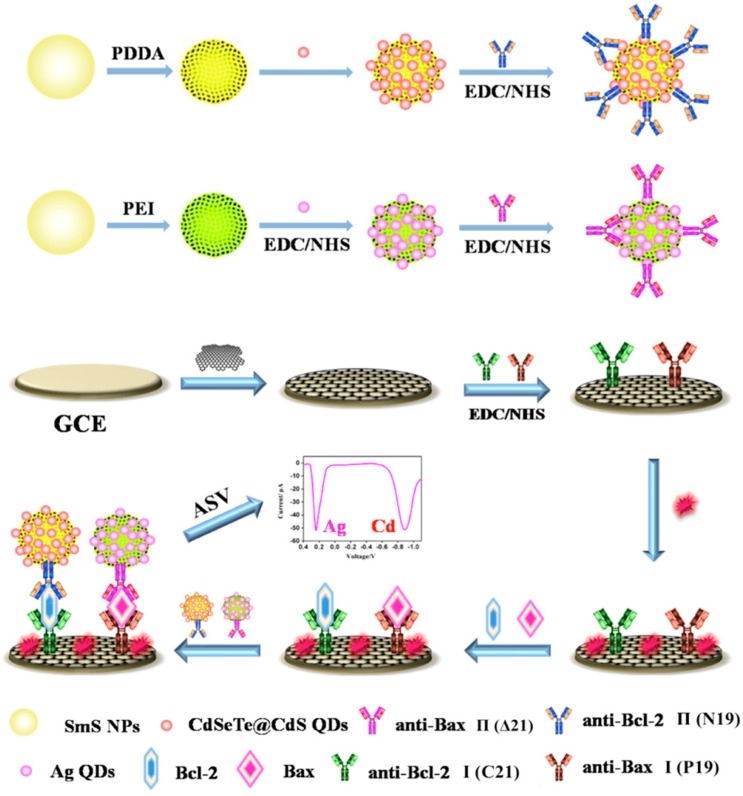
Fabrication of the dual-signal-marked electrochemical immunosensor for the detection of Bcl-2 and Bax. First, reduced graphene oxide (RGO) was used to modify glassy carbon electrode (GCE) to increase surface area and then linked antibodies I of Bcl-2, Bax. After bovine serum albumin (BSA) protein block the nonspecific adsorption sites, the electrode was immersed into the mixed antigens to capture the active Bcl-2 and Bax proteins. Finally, the electrode was reacted with antibody Π-targeted QDs and Ag nanoclusters as the signal probes, which were proportional to targets on the electrode. Reproduced with permission from [86].

Several electrochemical immunosensors while using QD are the focus on the detection of marker proteins on the surface of cell. For example, aiming to realize the simultaneous determination of EpCAM and GPC3 antigens on the surface of the human liver cancer cell line, M. Hui et al. [90] took advantage of CdTe QD-coated silica nanoparticles and ZnSe QD-coated silica nanoparticles to link the antibodies of EpCAM and GPC3, respectively. Chitosan-electrochemically reduced graphene oxide film (CS-GO) modified glassy carbon electrode (GCE) was used to immobilize the Ab1. The immunosensor showed superior reproducibility, accuracy, and stability. Similarly, Zhu et al. [91] reported a dual-signal-marked electrochemical immunosensor for the simultaneous detection of B-cell lymphoma 2 (Bcl-2) and Bcl-2-associated X protein (Bax), which are often used to monitor the apoptosis of tumor cells and to evaluate the cancer drug effect (Figure 12). CdSeTe@CdS QDs and Ag nanoclusters were used to connect with Ab2 of Bcl-2 and Bax, respectively. The electrochemical signal was amplified by mesoporous silica and graphene, which could provide abundant surface area for absorbance of probes and Ab1. Under anodic stripping voltammetry detection, Cd and Ag would be oxidized and produce currents under different potential, which represents the concentration of Bcl-2 and Bax, respectively.

## 3. Conclusions and Outlook

In conclusion, we reviewed the recent developments in nanomaterials-based electrochemical immunosensor, mainly including carbon, metal, and QD nanomaterials. With the continuous advancement of nanotechnology, the electrochemical immunosensor has been greatly developed. A compound to improve their water solubility, biocompatibility, and the ability to attach antibody, which were applied to modify the surface of electrode, mainly modified these nanomaterials. In addition, the nanomaterials are also used to label Ab2, which could enhance the sensitivity of electrochemical signal. Most reports have been realized the analysis in real sample while using electrochemical immunosensors. In clinical applications, electrochemical immunosensors could be utilized in the early detection of tumor and cancer biomarkers. 

Currently, researchers in the field seem to be focusing on the implementation of optimal performance limits for sensors and the lowest detection threshold at the small molecule level. However, we should also pay attention to electrochemical immunosensor not yet becoming the mainstream of practical application for some technical and commercial reasons. Long-time reproducibility and stability are needed to improve, because the electrode interface system is relatively complex. From Table 1 we could demonstrate that the reproducibility and stability of many prepared electrochemical immunosensors were not mentioned. It is a challenge in the functionalization of the electrode interface to improve the reproducibility and stability. Therefore, we need to consider at least three aspects when preparing an electrochemical immunosensor to overcome the problem of stability: (1) the immobilization of antibody is a pivotal step, because the antibody acts as the recognition element for antibody–antigen reaction. Selecting appropriate binding method is important, for example, the oriented antibody molecular layers are often used to improve the efficiency of binding. Huang et al. [92] reported an immunosensor for AFP detection, the current response of which could maintain about 96.2% of the original signal after 40 days. The good stability of the immunosensor was owed to the microenvironment that was provided by the layer-by-layer assembly film and the polymer PEDOT offered a stable substrate for the immobilization of antibodies. (2) Maintaining excellent stability of the probe is also vital for the electrochemical immunosensor. The functionalized performance of the nanomaterials that were used for adsorbing probe or the label of Ab2 should be stable and the method for linking is also required to be steady. For example, Zhang et al. [93] constructed an electrochemical sensor with good stability by using the Thi-CNTs sensing platform, which could prevent the leak of hydrophilic thionine from the electrode and avoid the addition of mediator to the solution, so the activity of the antibody could be kept for a long time. (3) How to actually apply the test results under ideal laboratory conditions to the detection of real samples and realize the demand of market for portable rapid detection technology.

Furthermore, the deep integration of electrochemical detection technology with nanotechnology, microfluidic technology, and other related technologies, including screen-printing and patterning, will definitely improve the performance of electrochemical immunosensors. The development of electrochemical immunosensor may present the trends of miniaturization and portability. Small and low-cost nanomaterials-based electrochemical sensing devices will be more popular in the near future.

## Figures and Tables

**Table 1:** Carbon Nanomaterials for electrochemical immunosensors.

Electrode Modification	Label	Analyte	Detection Range and LOD	Reproducibility	Stability	Reference
CP/RGO	Label-free	miRNA	Detection range: 1 fM–1 nM LOD: 5 fM	-	-	[42]
RGO-carbon nanotubes	Label-free	miRNA	LOD: 10 fM	-	-	[43]
streptavidin-functionalized NG	HRP	CEA	Linear range: 0.02–12 ng/mL LOD: 0.01 ng/mL	3.6%	95.8% (4 weeks)	[44]
PEI coated graphene	Label-free	GFAP	Linear range: 1 pg/mL–100 ng/mL	4.5%	-	[45]
SWNT forests	Carboxylated MWCNT-HRP	IL-6	LOD: 0.5 pg/mL	-	-	[48]
PGE/MWNT/Py	MNP	Insulin	LOD: 5 pM	-	-	[49]
MWCNTs/chitosan/GNDs	Label-free	NT-proBNP	Linear range: 0.01–100 pg/mL LOD: 3.86 fg/mL	3.3–5.9%	-	[50]
CS-AuNP/MWCNT/GO	Lactate oxidase	CHA	Linear range: 0.01–0.5 U/mL/0.5–100 U/mL LOD: 0.002 U/mL	7.6%	94.5% (2 weeks)	[51]
AuNPs-protein A	C_60_NPs-Au-PAMAM	Erythropoietin	Linear range: 0.01–80 mIU/mL LOD: 0.0027 mIU/mL	4%	86.3% (2 weeks)	[52]
CB@Ses-Qn	HRP	White spot syndrome virus	LOD: 990 nM	1.3%	-	[53]

**Table 2:** Metal Nanomaterials for electrochemical immunosensors.

Electrode Modification	Label	Analyte	Detection Range and LOD	Reproducibility	Stability	Reference
AuNPs/PGMA-g-PAN	Aq, FeC-COOH	PSA, AFP	Linear range: 10 pg/mL–100 ng/mL LOD: 2.2 pg/mL (PSA), 1.8 pg/mL (AFP)	-	-	[63]
Ag/MoS_2_/rGO	Label-free	CEA	Linear range: 0.01 pg/mL–100 ng/mL LOD: 1.6 fg/mL	<5%	100% (4 weeks)	[64]
Pd–Fe_3_O_4_-GS	SiO_2_	IgG	Linear range: 5 × 10^−6^–5 ng/mL LOD: 3.2 fg/mL	3.2%	100% (4 weeks)	[65]
CAP-MWCNTs	AuPd−CD	Adamantine	Linear range: 50 pg/mL–50 μg/mL LOD: 4.6 pg/mL	2.9%	93.5% (1 week)	[66]
MoS_2_/Au NPs	PtCu NPs/Cu_2_O@TiO_2_-NH_2_	Insulin	Linear range: 0.1 pg/mL–100 ng/mL LOD: 0.024 pg/mL	2.54–4.28%	-	[69]
IrOx	Label-free	AFP	Linear range: 0.05–150 ng/mL LOD: 20 pg/mL	<5.0%	14% (2 weeks)	[71]
Ag NPs	Co_3_O_4_@MnO_2_-Th	AFP	Linear range: 0.001–100 ng/mL LOD: 0.33 pg/mL.	<5.0%	86% (1 week)	[72]
TiO_2_-NGO/Au@Pd HSs	Label-free	HE4 Ag	Linear range: 40 fM–60 nM LOD: 13.33 fM	<2.3%	90.2% (4 weeks)	[73]
DpAu	Fc-Fc/β-CD/PAMAM−Au	PCT	Linear range: 1.80 pg/mL–500 ng/mL LOD: 0.36 pg/mL	3.7%	83.7% (4 weeks)	[74]
TiO_2_-NGO/Au@Pd HSs	Label-free	HE4 Ag	Linear range: 40 fM–60 nM LOD: 13.33 fM	<2.3%	90.2% (4 weeks)	[75]
D-Au film	Pt NWs@g-SBA-15/Thi	HBs Ag	Linear range: 10 fg/mL–100 ng/mL LOD: 3.3 fg/mL	1.31%	85.37% (4 weeks)	[76]
Dendritic gold-PCEPy	ALP	CTX	LOD: 1 ng/mL	-	-	[77]

**Table 3:** Quantum dot (QD) Nanomaterials for electrochemical immunosensors.

Electrode Modification	Label	Analyte	Detection Range and LOD	Reproducibility	Stability	Reference
-	CdSe/ZnS QD	BSA-OP	Linear range: 0.5–500 ng/mL LOD: 0.5 ng/mL	8.6%	-	[85]
ZrO_2_	QD	OP-BChE	Linear range: 0.1–30 nM LOD: 0.03 nM	4.5%	-	[86]
GS–PBSE	GS–QD	PSA	Linear range: 0.005–10 ng/mL LOD: 3 pg/ML	7.9%	88% (3 weeks)	[87]
AgNPs-PDA	CQDs-PEI-GO/AuNPs	CA15-3	Linear range: 0.005–10 ng/mL LOD: 3 pg/mL	2.3%	89.6% (4 weeks)	[88]
-	CdSe/ZnS QDs	tTG	LOD: 2.2 U/mL	5.9%	100% (4 weeks)	[89]
CS-GO	ZnSe QD-coated silica nanoparticles	EpCAM, GPC3 on the surface of Hep3B cell	Linear range: 5–1 × 10^6^ cells/mL LOD: 5 cells/mL	4.6% and 6.2%	>90% (2 weeks)	[90]
RGO	CdSeTe@CdS QDs, Ag nanoclusters	Bcl-2, Bax	LOD: 1 × 10^3^ cells	-	-	[91]
